# Methane-fuelled biofilms predominantly composed of methanotrophic ANME-1 in Arctic gas hydrate-related sediments

**DOI:** 10.1038/s41598-019-46209-5

**Published:** 2019-07-05

**Authors:** Friederike Gründger, Vincent Carrier, Mette M. Svenning, Giuliana Panieri, Tobias R. Vonnahme, Scott Klasek, Helge Niemann

**Affiliations:** 1 CAGE – Centre for Arctic Gas Hydrate, Environment and Climate, Department of Geosciences, UiT The Arctic University of Norway, Tromsø, Norway; 2 Department of Arctic and Marine Biology, UiT The Arctic University of Norway, Tromsø, Norway; 3 Department of Microbiology, College of Sciences, Oregon State University, Corvallis, OR USA; 4 Department of Marine Microbiology & Biogeochemistry, and Utrecht University, NIOZ Royal Netherlands Institute for Sea Research, ‘t Horntje, The Netherlands; 5 Department of Earth Sciences, Faculty of Geosciences, Utrecht University, Utrecht, The Netherlands

**Keywords:** Metagenomics, Marine microbiology, Environmental microbiology, Biofilms

## Abstract

Sedimentary biofilms comprising microbial communities mediating the anaerobic oxidation of methane are rare. Here, we describe two biofilm communities discovered in sediment cores recovered from Arctic cold seep sites (gas hydrate pingos) in the north-western Barents Sea, characterized by steady methane fluxes. We found macroscopically visible biofilms in pockets in the sediment matrix at the depth of the sulphate-methane-transition zone. 16S rRNA gene surveys revealed that the microbial community in one of the two biofilms comprised exclusively of putative anaerobic methanotrophic archaea of which ANME-1 was the sole archaeal taxon. The bacterial community consisted of relatives of sulphate-reducing bacteria (SRB) belonging to uncultured *Desulfobacteraceae* clustering into SEEP-SRB1 (i.e. the typical SRB associated to ANME-1), and members of the atribacterial JS1 clade. Confocal laser scanning microscopy demonstrates that this biofilm is composed of multicellular strands and patches of ANME-1 that are loosely associated with SRB cells, but not tightly connected in aggregates. Our discovery of methanotrophic biofilms in sediment pockets closely associated with methane seeps constitutes a hitherto overlooked and potentially widespread sink for methane and sulphate in marine sediments.

## Introduction

Microbial biofilms are structured multicellular aggregates of microbes that are enclosed in a matrix of mucoid self-produced extracellular polymeric substances (EPS, or exopolysaccharides)^1–3^. The structural features enhance the ability of microbial interactions within the biofilm, but also increase tolerance to adverse conditions and persistence against hostile environments. In natural marine ecosystems, biofilms are found on different types of surfaces ranging from animal skins and algae, various kinds of particles and aggregates, inert or bio-reactive minerals, and submerged constructions such as pilons or ship hulls^4,5^. Sediments are excellent substrates for microbial colonisation, providing nutrients and different types of electron acceptors and donors^6^. However, knowledge on the formation of biofilms in pockets, cracks or fractures within the sediment matrix is limited, and it is unclear how extensive such subsurface microbial aggregations are^7^ along with their potential role as a geological sink for methane and sulphate.

A globally important microbial process in anoxic marine sediments is the anaerobic oxidation of methane (AOM) with sulphate as the terminal electron acceptor^8,9^:

$${{\rm{CH}}}_{4}+{{\rm{SO}}}_{4}^{2-}\to {{\rm{HCO}}}_{3}^{-}+{{\rm{HS}}}^{-}+{{\rm{H}}}_{2}{\rm{O}}$$

This process is mediated by anaerobic methanotrophic archaea (ANME-1, -2, -3), typically with partner sulphate-reducing bacteria (SRB) of the *Desulfosarcina/Desulfococcus*-related clade Seep-SRB1 (ANME-1, -2) or *Desulfubulbus* sp. (ANME-3)^10–15^. Because AOM communities depend on the availability of sulphate and methane, they normally occupy (and shape) sulphate-methane transition zones (SMTZ), which are located in reduced sediment layers. These layers can be found several tens to hundred meters below the sediment surface, but at cold seeps (such as Gas Hydrate Pingos - GHPs), elevated methane fluxes lead to a shallower SMTZ in near-surface sediments^16–19^. Indeed, the abundance of AOM communities was generally found to peak at depth of SMTZ^12^. So far, the buildup of biofilms/aggregations primarily comprising ANME/SRB biomass has only been observed in the anoxic waters of the Black Sea where AOM biomass may form reef like structures^20,21^. In addition, at two sites in fractured gas hydrate-bearing sediments of the Pacific and Indian Ocean, Briggs, *et al*.^7^ found AOM communities dominating biofilms at depth of the SMTZ. However, our knowledge on AOM community distribution is primarily based on sediment core analyses, which typically does not resolve horizontal variations of microbes clumped in spatially confined biofilms in sediment pockets/cracks^22^.

In this study, we report on the finding of macroscopically visible biofilms that we found in pocket-like features in reduced, methane-rich sediments from a GHP area south of the Svalbard archipelago in the Arctic Ocean. Furthermore, we describe the exceptional microbial community composition, which differ strongly from any other environmental biofilm investigated to date.

## Material and Methods

### Sample collection and processing

**Figure 1:**
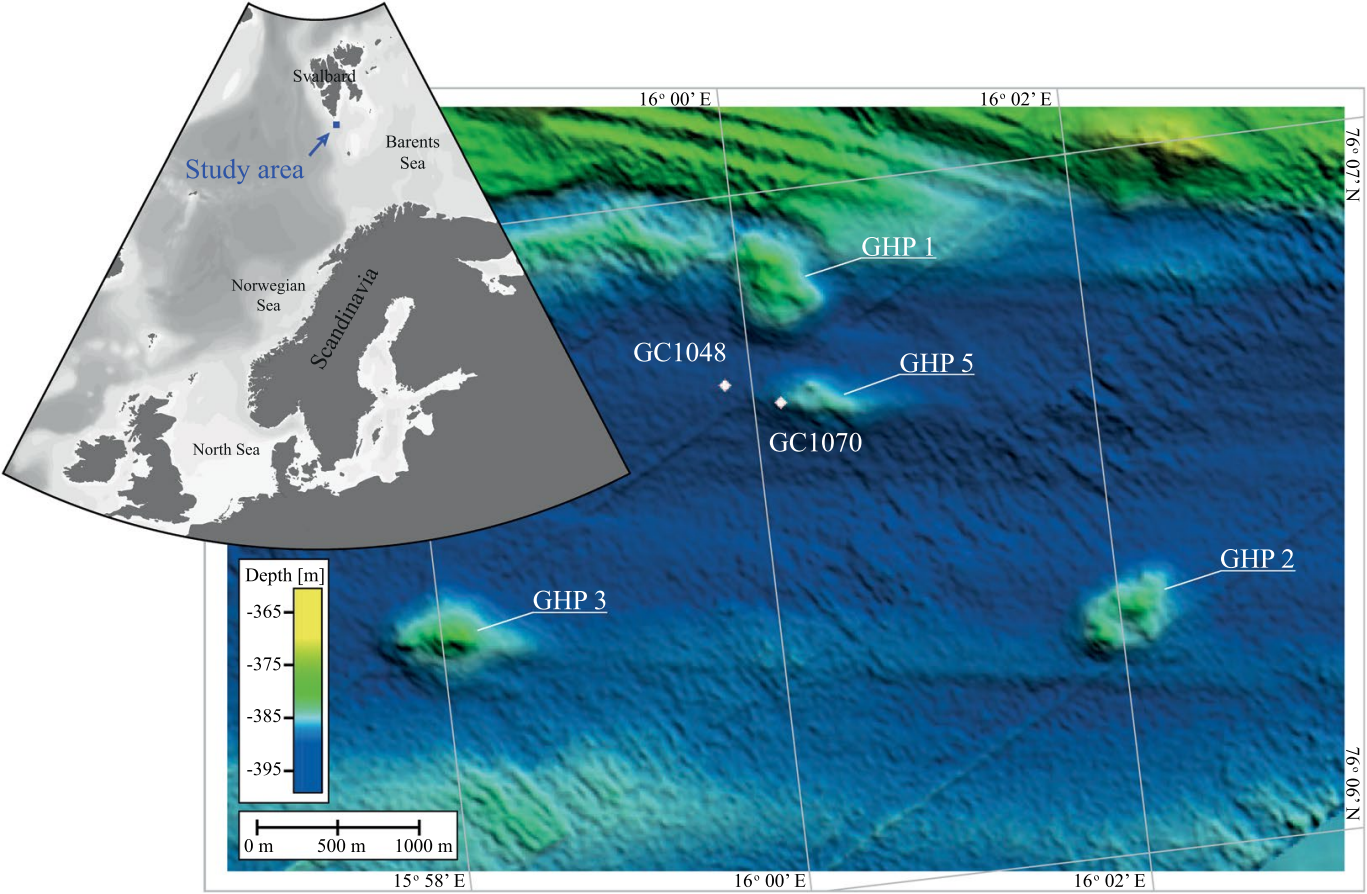
Regional bathymetry and the geographic core positions of GC1048 (76° 06.737N; 15° 59.845E) and GC1070 (76° 06.703N; 16° 00.162E) (white diamonds) at Storfjordrenna south of Svalbard Archipelago. Names to the gas hydrate pingos (GHPs) are given.

**Figure 2:**
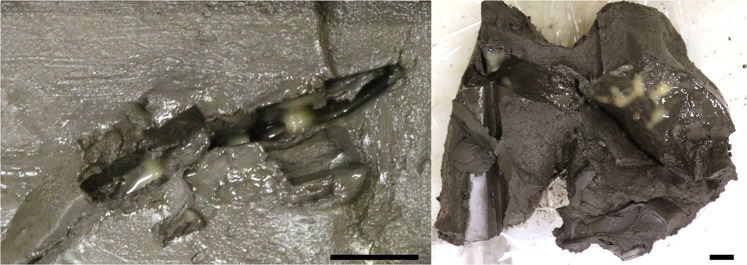
Sediment core GC1048 with biofilm pocket after retrieval, cutting the core into half and sampling. Scale bars = 1 cm.

Sediments were collected with a gravity corer (GC) during a research expedition (CAGE16-5) with R/V *Helmer Hanssen* in June 2016 to the GHP area at Storfjordrenna, which is south of the Svalbard archipelago in the north-western Barents Sea (Storfjordrenna Trough Mouth Fan, ~390 m water depth). The area is characterized by five GHPs. Four of them show active gas discharge in form of numerous gas flares rising up to 20 m below sea level^23^. At GHPs with active methane seepages, shallow gas hydrate layers were discovered, some of them only 40 cm below sea floor^23^. GHP 5 is proposed to be in a post-active phase of seepage^16^, being the one without observed flare activity and gas hydrate recovery. We recovered one sediment core (GC1070; length: 326 cm) from the rim of GHP 5 and a second one (GC1048; length: 335 cm) ~350 m to the west of the edifice (Fig. 1). Immediately after recovery, the cores were cut into 100 cm sections, split longitudinally and sub-sampled in a cold room. In both cores, we found pockets of 4–5 cm length in the sediment matrix filled with a macroscopically visible slimy yellow-greenish biofilm (Fig. 2). Subsamples from these biofilms were taken with a sterile spatula. We obtained a pure biofilm sample from GC1048 (i.e. no sediment particles were visible in the sample), while the sample collected from core GC1070 contained some visible sediment admixture. The samples were transferred into sterile 2-ml Eppendorf tubes. Samples for DNA analyses were stored at −20 °C. Samples for microscopy studies were fixed in 4% (w/v) formaldehyde solution as described by Pernthaler, *et al*.^24^ and stored in 1:1 mix of PBS / ethanol at −20 °C. After the cruise, sedimentological descriptions were performed in our home laboratory. For the examination of the core’s sediments, smear slides were prepared from the sediments close to the biofilm following the methods described by Marsaglia *et al*.^25^ and observed with a petrographic microscope.

### Fluorescence-*in-situ*-hybridization

For fluorescence-*in-situ*-hybridizations (FISH), 50 µl of fixed biofilm sample was diluted in 1 ml 1 × PBS, filtered on a 25 mm polycarbonate filter (0.2 µm pore size) and embedded in 0.2% w/v Agarose. FISH was done using double-labelling-of-oligonucleotide-probes (DOPE; Stoecker, *et al*.^26^) for *Archaea* (ARCH915; Stahl and Amann^27^) and *Desulfobacteraceae* (DSS658; Mußmann, *et al*.^28^) synthesized by biomers.net GmbH (Ulm/Donau, Germany). The antisense probe NON338^29^ was used to test for unspecific staining for the given formamide concentrations. The probes were labelled at the 5′ and 3′ end with Cyanine 3 (ARCH915) and 6-FAM (DSS658, NON338). Hybridizations were done in accordance to published work^30^ with 3 h hybridization with DSS658 (50% formamide) followed by ARCH915 (0% formamide). The NON338 probe was incubated for 3 h (0% formamide). After DOPE FISH, the samples were counterstained with DAPI as described by Glöckner *et al*.^31^. Imaging was done with a confocal laser scanning microscope (Axio Observer LSM800, Carl Zeiss Microscopy GmbH, Jena, Germany) using a Plan-Apochromat 63x/1.40 Oil M27 objective. Emission and detection wavelengths were 561 and 535–700 nm for Cy3, 488 and 450–545 nm for 6-FAM, and 405 and 400–600 nm for DAPI.

### DNA extraction, 16S rRNA gene amplification and sequencing analysis

DNA from 10 mg of biofilm sample from core GC1048 and 245 mg from core GC1070 was extracted in a clean laminar flow hood using a Qiagen DNeasy PowerSoil kit according to the manufacturer’s instructions.

For the amplification of 16S rRNA genes, we used the degenerated primer sets A519F (5′-CAGCMGCCGCGGTAA)^32^ and A906R (5′-CAATTCMTTTAAGTTTC)^33^ for *Archaea* and Bakt_341F (5′-CCTACGGGNGGCWGCAG) and Bakt_805R (5′-GACTACHVGGGTATCTAATCC) for *Bacteria*^34^. 16S rRNA gene amplification and sequencing were carried out by IMGM Laboratories GmbH (Martinsried, Germany). Cluster generation and bidirectional sequencing by synthesis was performed on Illumina MiSeq next generation sequencing system (Illumina, CA, USA) using reagents kit 500 cycles v2 under the control of MiSeq Control Software v2.5.0.5. Obtained reads were meticulously processed following a modified version of the USEARCH protocol (http://drive5.com/usearch/manual/uparse_pipeline.html; Supplementary Information S1). Taxonomy was assigned using the SILVA database release 132^35^. Non-16S rRNA gene sequences as well as OTUs containing single sequence or best assigned to non-targeted domains were removed. Nucleotide sequences have been deposited at SRA database (https://www.ncbi.nlm.nih.gov/sra) as BioProject with accession number PRJNA506542.

Furthermore, phylogenetic analyses of the abundant OTUs associated with the ANME-1 group and *Desulfobacteracae* were conducted to accurately assess their evolutionary origin from our Illumina MiSeq reads^36^. For this, we selected 19 ANME-1 (min. length: 1300 bp) and 32 *Desulfobacteraceae* sequences (min length: 807 bp) from published phylogenies to form a phylogenetic tree for each taxonomic group. Sequences were aligned using MUSCLE^37^ implemented in MEGA 7 and a best-scoring maximum likelihood phylogenetic tree was built in Randomized Axelerated Maximum Likelihood (RAxML; Stamatakis^3^) using the General Time Reversible (GTR) Gamma model. Thereafter, shorter reads of the OTUs collected from biofilm in core GC1048 and GC1070 were aligned to the previously selected sequences and were placed on the built phylogenetic trees using the Evolutionary Placement Algorithm implemented in RAxML^3,36^. Resulting trees were visualized and annotated in Interactive Tree Of Life^38^.

## Results and Discussion

**Figure 3:**
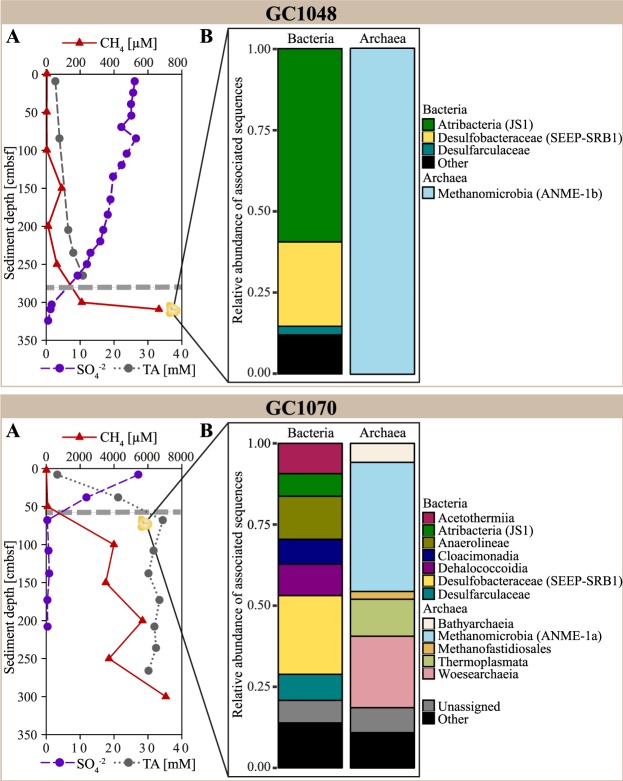
Data from sediment core GC1048 and GC1070. In each box, (**A**) Depth profile of concentrations of alkalinity (TA), sulphate (SO_4_^−2^), and dissolved methane (CH_4_)^16^. The dashed grey line indicates the SMT depth of each core. The position of the biofilms is indicated as yellow symbol (GC1048: 305 cmbsf, GC1070: 68 cmbsf). Symbol size do not represent the actual size of biofilm. (**B**) Sequence-based relative abundances of bacterial and archaeal 16S rRNA genes. ‘Other’ includes taxa with less than 1% relative sequence abundance within the sequence data set. ‘Unassigned’ includes sequences that could not be assigned to a taxonomic group within their respective domain.

At GHP 5 and its close vicinity, we recovered two sediment cores (Fig. 1) comprising pockets in the sediment matrix that were filled with a macroscopically visible, slimy, yellow-greenish biofilm (Fig. 2). Pockets/biofilms of 4–5 cm length were found at 305 cmbsf within core GC1048 and at 68 cmbsf within core GC1070 (visualized as yellow symbol in Fig. 3A), which is in both cases less than ten centimetres below the depth of the SMTZ^16^. The cores were composed of glacigenic sediments, with hemipelagic grey mud comprising variable amounts of ice-rafted debris. Ice-rafted debris were deposited during several phases of extensive iceberg production. In both cores, the sediment horizons where the biofilms were found were characterized by laminated hemipelagic grey mud and silts mainly composed of quartz, carbonates, feldspar, and clay minerals. Besides the pockets, we did not observe any other sedimentological feature or sediment colour changes that could indicate a preferential site for biofilm formation.

### Biofilm microbial composition

For microbial diversity analysis of the two biofilm samples, we processed 82654 archaeal and 74083 bacterial read pairs. In total, reads clustered into 136 archaeal and 238 bacterial OTUs.

**Figure 4:**
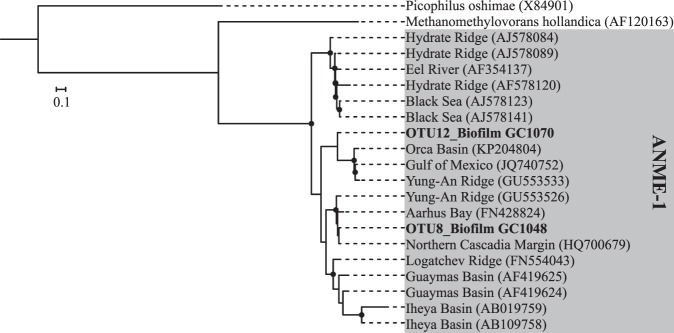
Phylogenetic tree showing evolutionary connections of the dominant OTUs representing biofilm 16S rRNA gene sequences to selected reference sequences of uncultured archaea of ANME-1 clades. Boldface type indicates the sequences obtained in this study. The tree was calculated by using RAxML algorithm. Biofilm sequences (~500 bp) were inserted by using EPA. Black dots at branches represent bootstrap values higher than 50. The bar indicates 10% estimated phylogenetic divergence.

**Figure 5:**
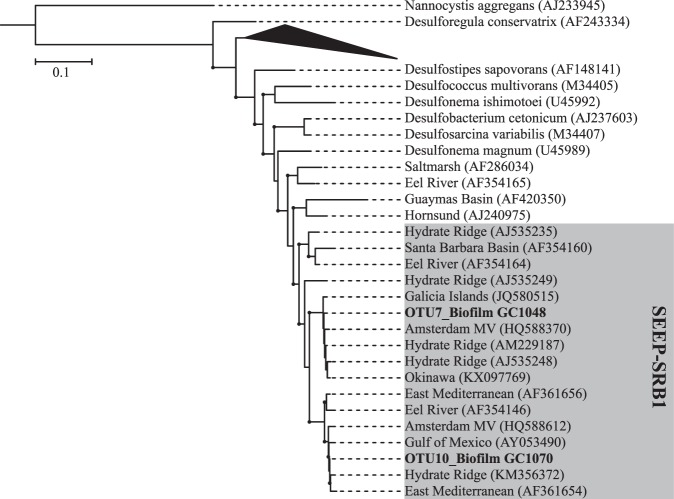
Phylogenetic tree showing evolutionary connections of the dominant OTUs representing biofilm 16S rRNA gene sequences to selected reference sequences of *Desulfobacteraceae* from the environment and isolated strains. Boldface type indicates the sequences obtained in this study. The tree was calculated by using the RAxML algorithm. Biofilm sequences (~500 bp) were inserted by using EPA. Black dots at branches represent bootstrap values greater than 50. The scale bar indicates 10% estimated phylogenetic divergence.

The microbial community in the biofilm from GC1048 showed an extremely low diversity (Shannon diversity index of 0.001 and 1.22 for *Archaea* and *Bacteria*, respectively; see Supplementary Table S1). All archaeal sequences clustered exclusively into one OTU (OTU8) that was associated with the anaerobic methanotrophic archaea (ANME) clade ANME-1b (Fig. 4). Among the most abundant bacterial groups, we found members closely related to the typical partner SRB of ANME-1, i.e. *Desulfobacteraceae* clustering into the SEEP-SRB1 clade (26% of all bacterial sequences; Fig. 5). Additional 3% of bacterial sequences were identified as *Desulfatiglans* (*Desulfarculaceae*), which is another common SRB in methane seep environments often associated with ANME^39–41^. Together with the vertical positioning of the biofilm close to the SMTZ, our sequence analyses suggest that the biofilm was predominantly involved in AOM and was mostly comprised of AOM-related biomass.

ANME-1-dominated biofilms in natural environments are very rare. Michaelis, *et al*.^20^ reported on microbial mat biomass from microbial reefs in the Black Sea that was comprised of only one archaeal population (belonging to ANME-1) forming consortia with partner SRB of the *Desulfosarcina/Desulfococcus* group. Treude *et al*.^21^ described similar mat structures in sediments from the Black Sea. The only other finding of biofilms in ‘regular’ ocean sediments was made by Briggs, *et al*.^7^, who described ANME-dominated biofilms in fractures at depth of the SMTZ at the northern Cascadia Margin and the Indian Ocean. In those biofilms, the ANME-1 clade was identified as the most abundant taxon of a more diverse archaeal community, which included members of *Thermoplasmatales* and *Methanosarcinales*. Thus, our findings of an archaeal community in the biofilm from GC1048, which was exclusively comprised of members of the ANME-1b clade is unique and not comparable to any other environmental biofilm found so far.

In addition to AOM-related biomass in core GC1048, we detected members of *Atribacteria* (JS1 clade, 60% of all bacterial sequences). The JS1 clade co-occur especially predominant with organic carbon replete, and methane-rich conditions in anaerobic marine sediments. It has been suggested that this group has an anaerobic heterotrophic lifestyle^42,43^ rather than being direct involved in AOM^44,45^. However, the metabolic potential of this uncultured clade and its relation to AOM remains unconstrained, because knowledge on the metabolic potential of JS1 is based on single-cell amplified genome analyses^42^. Other bacterial taxa found in biofilm from core GC1048 were *Bacteroidetes* (4%), *Spirochaetes* and uncultured TA06 clade (3% each; Fig. 3B).

Similar to the biofilm from core GC1048, the archaeal community of the biofilm within core GC1070 was dominated by members of the ANME-1 clade (43%), though, in contrast to GC1048, ANMEs in GC1070 most probably belonged to subgroup a, rather than subgroup b. Furthermore, contrary to GC1048, the microbial diversity within the GC1070 biofilm was higher (Shannon diversity index of 2.28 and 3.29 for *Archaea* and *Bacteria*, respectively). In addition to ANME-1a, we identified members of *Woesearchaeia* (24%), *Thermoplasmata* (MBG-D and DHVEG-1; 14%), *Thermococci* (*Methanofastidiosales;* 3%) and *Bathyarchaeia* (6%). Among members of the domain *Bacteria*, we found the AOM-associated taxa SEEP-SRB1 (24%) and *Desulfatiglans* (8%). Other abundant taxa were *Chloroflexi* (23%, *Anaerolineae/Dehalococcoidia*), *Acetothermia* (9%), *Atribacteria* (JS1 clade, 7%), *Cloacimonetes* (6%), and *Planctomycetes* (2%) (Fig. 3B). We suggest that the higher diversity is caused by the admixture of sediments (and sediment-associated microbes) to the biofilm sample. Indeed, representatives belonging to *Anaerolineae*, *Dehalococcoidia*, *Atribacteria*, *Woesearchaeia*, and MBG-D and DHVEG-1, commonly encountered at methane seeps, can be related to organic matter degradation (e.g. Inagaki, *et al*.^45^, Pop Ristova, *et al*.^46^, Nunoura, *et al*.^47^, Trembath-Reichert, *et al*.^48^, Inagaki, *et al*.^49^, Cruaud, *et al*.^50^). *Woesearchaeia* are often found in marine environments with high organic matter content^47,51^, but are also linked to symbiotic or parasitic lifestyles based on small genome sizes and limited metabolic capabilities^52^. However, at our sampling site, neither siboglinid tubeworms nor any other chemosynthetic macrofauna species were observed^53^. MBG-D and DHVEG-1, *Anaerolineae*, and *Dehalococcoidia* might play a major role in protein, amino acid and fatty acid re-mineralization^50,54^; *Dehalococcoidia* could also mediate reductive dehalogenation and potentially reduce sulphate^55–57^. All those substrates are probably available (at least to some degree) at the sediment horizon where the biofilm within GC1070 was found.

### Biogeochemical functioning of biofilm microbes

According to the methane and sulphate concentration profiles (Fig. 3A)^16^, the SMTs were likely located at ~300 cmbsf in core GC1048 and ~60 cmbsf in GC1070, i.e. less than ten centimetres above to where we discovered the biofilms. Both cores are characterized by steady-state sulphate-methane dynamics that ensures a consistent supply of both sulphate and methane at the SMTZ^16^. However, the differential depths of the SMTZ in the two cores suggest dissimilar methane fluxes. In GC1048, the SMTZ appeared to be deeper compared to GC1070 implying a lower methane flux in GC1048 than in GC1070.

The co-localisation of the biofilm and the SMTZs together with the prevailing presence of ANME-1 archaea and potential partner SRB in the biofilm samples, thus suggests that these microbes mediate sulphate-dependent AOM^12^.

**Figure 6:**
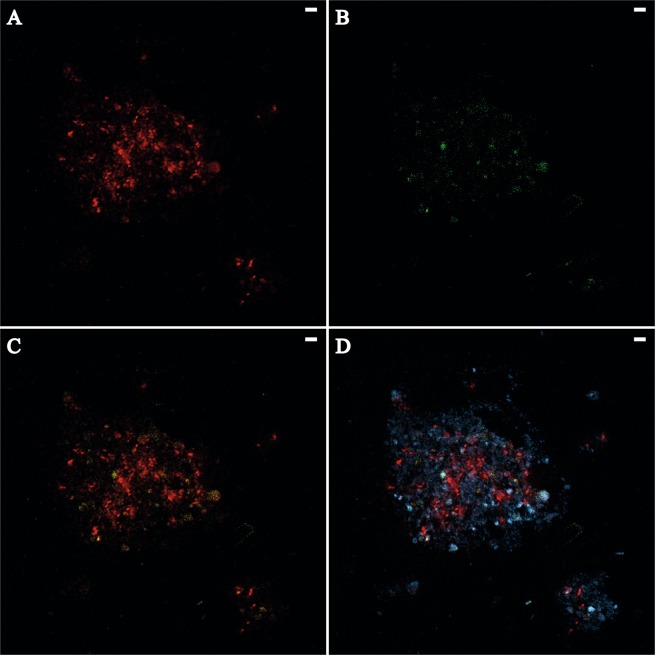
Confocal laser scanning micrographs of *Archaea* and sulphate-reducing bacteria (SRB) in biofilm from GC1048 visualized by FISH. Scale bars = 2 µm. (**A**) Archaeal cells (probe ARCH915 labelled with Cy3 [red]. (**B**) SRB belonging to *Desulfobacteraceae* (probe DSS658 labelled with 6-FAM [green]). (**C**) Overlay of image A and B (probe ARCH915 and DSS658). (**D**) Overlay of image A and B and nucleic acids stained with DAPI [blue].

We analysed the cellular structure of the AOM biofilm community from GC1048 by confocal laser scanning microscopy. Images revealed numerous globular tight cell clusters of sulphate-reducing *Desulfobacteraceae* ranging 1–3 µm in diameter (Fig. 6B), but also loose cell formations. Archaeal cells (identified as ANME-1b by sequencing analysis) formed many small tight globular clusters (<1 µm) as well as patches of loose cell formations (Fig. 6A). Similar to previous findings^58,59^, we found ANME-1b cells as multicellular strands/chains with length of several tens of micrometres. SRB cells seemed to be loosely associated with some of the multicellular ANME-1b strands and patches (Fig. 6C). In our biofilm sample, we did not observe any direct cell-to-cell contact of SRB and ANME-1b cells as shown for ANME-2/DSS aggregates^10,59,60^ or the shell-type consortia of ANME-3 and *Desulfobulbus* spp.^11,61^. Previous studies showed that in sediments, ANME-1 may exist as single cells or as mono-specific chains or clusters without direct, physical association of partner SRB^59–62^ raising the question if ANME-1 could also mediate AOM alone^12^, as found in some cases for ANME-2^15^.

ANME-1a and ANME-1b subgroups only contain uncultivated strains and their phylogenetic distance to each other shows sequence similarity values of <96% based on 16S rRNA genes^60^. In our biofilms, OTU8 clustered confinently into subclade 1b, while OTU12 seems to be closer associated to subclade 1a although that its assignment to one of the two clades is less certain. Still, the phylogenetical assignments of our OTUs to either ANME-1a and 1b is supported by the reference sequences from methane-rich environmental samples shown in Fig. 4. The environmental factors for selecting the two subgroups are still unknown. Both subgroups have been found at methane seeps^12^. The dominance of ANME-1 archaea in or below a SMTZ located some meters below seafloor has also been reported from sediments from a North Sea gas seep (up to 2.5 mM CH_4_; Niemann, *et al*.^63^), the Santa Barbara Basin (>3 mM CH_4_; Harrison, *et al*.^64^), the Sea of Japan (~1.8 mM CH_4_; Yanagawa, *et al*.^65^), all of which are characterized by relatively high methane but rather low sulphate availability. Similarly, ANME-1b archaea were found to dominate highly sulphate-depleted sediments at Haima cold seeps in the South China Sea^66^. Moreover, flow chamber incubation experiment have shown that ANME-1 archaea are more active at high methane flow rates compared to ANME-2, which are only minimally affected by increased flow rates^67^. Only at the Black Sea microbial mat reefs, an ANME-1-dominated AOM community was found in an environment with high methane and high sulphate supply^20^. On the other hand, ANME-1 archaea were also found to dominate highly saline environments with moderate sulphate and rather low methane concentrations at a mud volcano in the Gulf of Cadiz (<0.6 mM CH_4_; Maignien, *et al*.^62^) and in hypersaline environments of the Gulf of Mexico (<0.2 mM CH_4_; Lloyd, *et al*.^68^). While high methane fluxes, low sulphate concentrations and hypersaline conditions may thus select for ANME-1 clades, the ecological niches of ANME-1a vs. ANME-1b are not well constrained. We can thus only speculate why ANME-1a and ANME-1b clades separately dominate each of the biofilms. Nevertheless, our findings suggest that ANME-1b, when compared to ANME-1a, seems to prevail in deeper, more sulphate-depleted sediments at sites of low methane flux.

## Summary and Conclusion

In this paper, we report on biofilms that occupy sediment pockets located just below the SMTZ in two cores recovered from a cold seep area characterized by steady-state sulphate-methane dynamics. Both biofilms were dominated by AOM communities comprised of members of ANME-1 and SEEP-SRB1, which have only been reported once previously in the literature^7^. Furthermore, one of the biofilms was exclusively comprised of ANME-1b archaea, which built multicellular strands and patches only loosely associated with SRB cells. This raises the general question if ANME-1 can mediate AOM alone without any partner SRB. The second biofilm was characterized by a higher microbial diversity, possibly caused by admixture of the biofilm sample with surrounding sediments, but with ANME-1a as the dominant archaeal taxa. It remains ambiguous as to which environmental factors control the selection of subgroups ANME-1a or ANME-1b in natural environments. This investigation suggests that ANME-1b, in comparison with ANME-1a, appear to prevail in deeper, more sulphate-depleted sediments with a lower methane flux. Our findings also support the proposition that sub-seafloor sediment pockets and micro-fractures in a methane-related advective system promote AOM biofilm formation by providing pockets and conduits within sediment matrices where methane potentially accumulates or flows through. This constant supply of methane supports the development of AOM communities, which, over time, form biofilms^22^. Sediment pockets and micro-fractures could be more extensive at methane seeps than previously assumed.

## Supplementary information

Supplementary Material

## Data Availability

Nucleotide sequences have been deposited at SRA database (https://www.ncbi.nlm.nih.gov/sra) as BioProject with accession number PRJNA506542. Detailed information on sequencing read processing workflow are available in the Supplementary Information S1.
