# Supplementary material for: Methane-fuelled biofilms predominantly composed of methanotrophic ANME-1 in Arctic gas hydrate-related sediments
Source: Sci Rep. 2019 Jul 5;9:9725. doi: 10.1038/s41598-019-46209-5 (PMC6611871; doi:10.1038/s41598-019-46209-5)
Supplement: Supplementary file 1 — Supplementary Material [file 41598_2019_46209_MOESM1_ESM.pdf]

Supplementary Material

Methane-fuelled biofilms predominantly composed of methanotrophic ANME-1 in Arctic gas hydrate-related sediments

Friederike Gründger, Vincent Carrier, Mette M. Svenning, Giuliana Panieri, Tobias R. Vonnahme, Scott Klasek, Helge Niemann

1 Supplementary Information S1: Sequencing read processing workflow

Sequencing reads were processed following a modified version of the USEARCH protocol (http://drive5.com/usearch/manual/uparse_pipeline.html).

1. Prior to sequence processing through the pipeline, the quality score per base pair position was visually analysed using command line tools from FASTX-Toolkit (http://hannonlab.cshl.edu/fastx_toolkit/index.html).

```
For file in *.fastq
do fastx_quality_stats -Q33 -i $file -o $file.fastq_stats.txt
fastq_quality_boxplot_graph.sh -i $file.fastq_stats.txt -o $file.fastq_stats.png
done
```

2. Sequencing reads were merged using command line tool from USEARCH v10.0.240 (Edgar and Flyvbjerg, 2015). Minimum overlapping length and total merged length of the sequence were selected based on the length covered by the pair reads. The quality thresholds were determined from the quality scores overview from step 1.

```
usearch -fastq_mergepairs *R1* -fastqout pooled.merged.fastq -fastqout_notmerged_fwd
pooled.R1.unmerged.fastq -fastq_minqual -fastqout_notmerged_rev pooled.R2.unmerged.fastq -
relabel @ -fastq_trunctail -fastq_minmergelen -fastq_minovlen -fastq_maxdiffs -fastq_pctid
```

Parameters selected:
-fastq_minqual (Archaea: 10, Bacteria: 10)
-fastq_trunctail (Archaea: 20, Bacteria: 24)
-fastq_minmergele (Archaea: 250, Bacteria: 380)
-fastq_minovlen (Archaea: 16, Bacteria: 16)
-fastq_maxdiffs (Archaea: 10, Bacteria: 10)
-fastq_pctid (Archaea: 80, Bacteria: 80)

3. Primers were removed using command line tool from USEARCH v10.0.240 (Edgar, 2010). Paired reads were truncated according to the length of their primers.

```
usearch -fastx_truncate pooled.merged.fastq -stripleft -stripright -fastqout
pooled.merged.stripped.fastq
```

Parameters selected:
-stripleft (Archaea: 15, Bacteria: 17)
-stripright (Archaea: 17, Bacteria: 21)

4. Paired reads were quality filtered using command line tool from USEARCH v10.0.240 (Edgar and Flyvbjerg, 2015).

usearch -fastq_filter pooled.merged.stripped.fastq -fastq_maxee 1.0 -fastq_minlen 300 -fastqout pooled.merged.stripped.filtered.fastq

Parameters:
-fastq_maxee (Archaea: 1.0, Bacteria: 1.0) #Selected based on the authors' recommendations (Edgar and Flyvbjerg, 2015)
-fastq_minlen (Archaea: 300, Bacteria: 300) #Selected based on the amplicon size

5. Prior to forming OTUs, reads in libraries were de-replicated using command line tool from USEARCH v10.0.240 (Edgar, 2010). At this step, all unique sequences were discarded.

usearch-fastx_uniques pooled.merged.stripped.filtered.fastq -fastqout pooled.merged.stripped.filtered.uniques.fastq -fastaout pooled.merged.stripped.filtered.uniques.fasta -sizeout -relabel Uniq -minuniquesize 2

6. OTUs were formed using the UPARSE-OTU algorithm. The algorithm discards chimeric sequences and cluster sequences at = or > 97% identity (Edgar, 2013). A minimum of two sequences per OTU is set to retain the OTU.

usearch -cluster_otus pooled.merged.stripped.filtered.uniques.fasta -otus pooled.merged.stripped.filtered.uniques.otus.fasta -relabel OTU -uparseout log.txt -minsize 2

7. OTUs formed, a OTUs table was built per domain of life using command line tool from USEARCH v10.0.240 (Edgar, 2010).

usearch -otutab pooled.merged.stripped.fastq -otus pooled.merged.stripped.filtered.uniques.otus.fasta -strand both -id 0.97 -otutabout otu_table.txt -biomout otu_table.json -sizeout -matchedfq matched.reads.fastq -notmatchedfq unmatched.reads.fastq -mapout UPARSE_mapout.txt -mothur_shared_out mothur_UNOISE.txt -sizeout

8. Once the OTU tables were formed, taxonomic assignment was made using command line tool from Mothur (Schloss et al., 2009). Thereafter, the taxonomy column was added to their respective OTU tables using the Biom Format (McDonald et al., 2012).

mothur"#classify.seqs(fasta=pooled.merged.stripped.filtered.uniques.otus.fasta, reference=silva.nr_v132.align, taxonomy=silva.nr_v132.tax, cutoff=75)"

biom add-metadata --sc-separated taxonomy --observation-header OTUID,taxonomy --observation-metadata-fp pooled.merged.stripped.filtered.uniques.otus.nr_v132.wang.taxonomy -i otu_table.biom -o otu_table.taxonomy.biom

References

Edgar, R.C. Search and clustering orders of magnitude faster than BLAST. *Bioinformatics* 26(19), 2460-2461 (2010).

Edgar, R.C. UPARSE: highly accurate OTU sequences from microbial amplicon reads. *Nature Methods* 10, 996 (2013).

Edgar, R.C., and Flyvbjerg, H. Error filtering, pair assembly and error correction for next-generation sequencing reads. *Bioinformatics* 31(21), 3476-3482 (2015).

McDonald, D., et al. The Biological Observation Matrix (BIOM) format or: how I learned to stop worrying and love the ome-ome. *GigaScience* 1(1), 7 (2012).

Schloss, P.D., et al. Introducing mothur: Open-Source, Platform-Independent, Community-Supported Software for Describing and Comparing Microbial Communities. *Applied and Environmental Microbiology* 75(23), 7537-7541 (2009).

2 Supplementary Tables and Figures

Supplementary Table S1. Overview of the number of observed $OTU_{0.03}$ (sobs; i.e. observed richness), estimated richness (Chao1), Shannon diversity, Pilou`s evenness index; data were standardized by subsampling use the size of the smallest library (*Bacteria* = 4000, *Archaea* = 9000).

	Archaea		***Bacteria***	
	GC1048	GC1070	GC1048	GC1070
sobs	2	52	19	220
Chao1	2	55	19	253
Shannon	0.0011	2.28	1.22	3.29
Pilou	0.0016	0.58	0.41	0.61

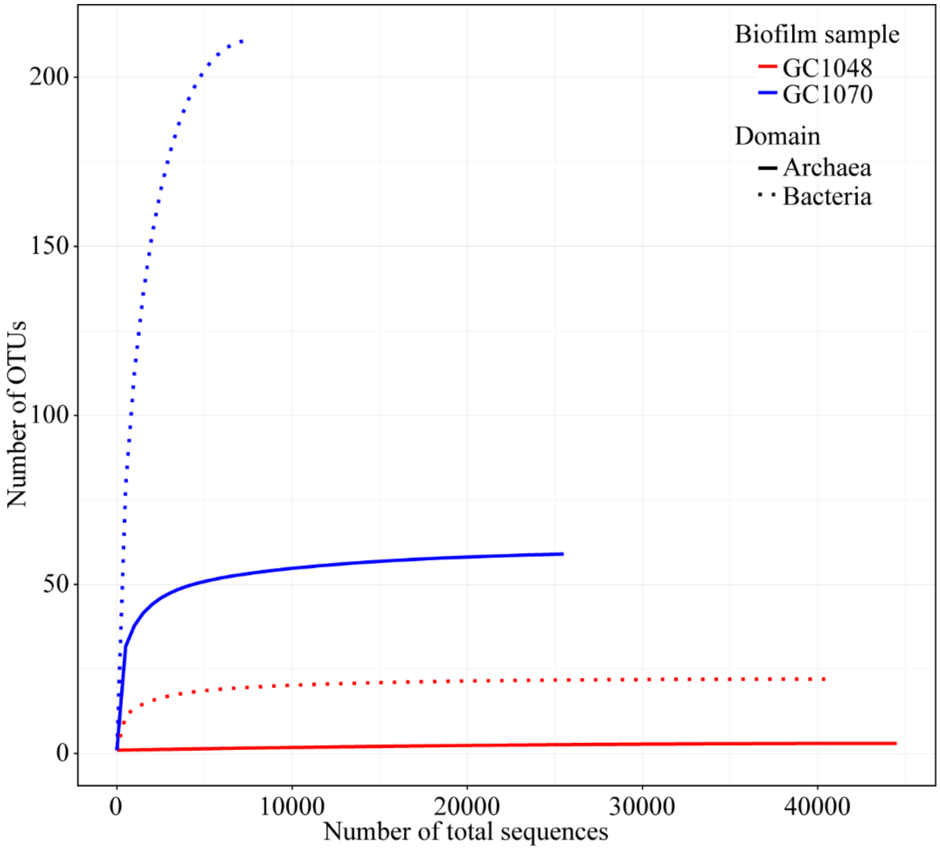

Supplementary Figure S1. Calculated rarefaction curves of observed OTUs from biofilms from GC1048 and GC1070.

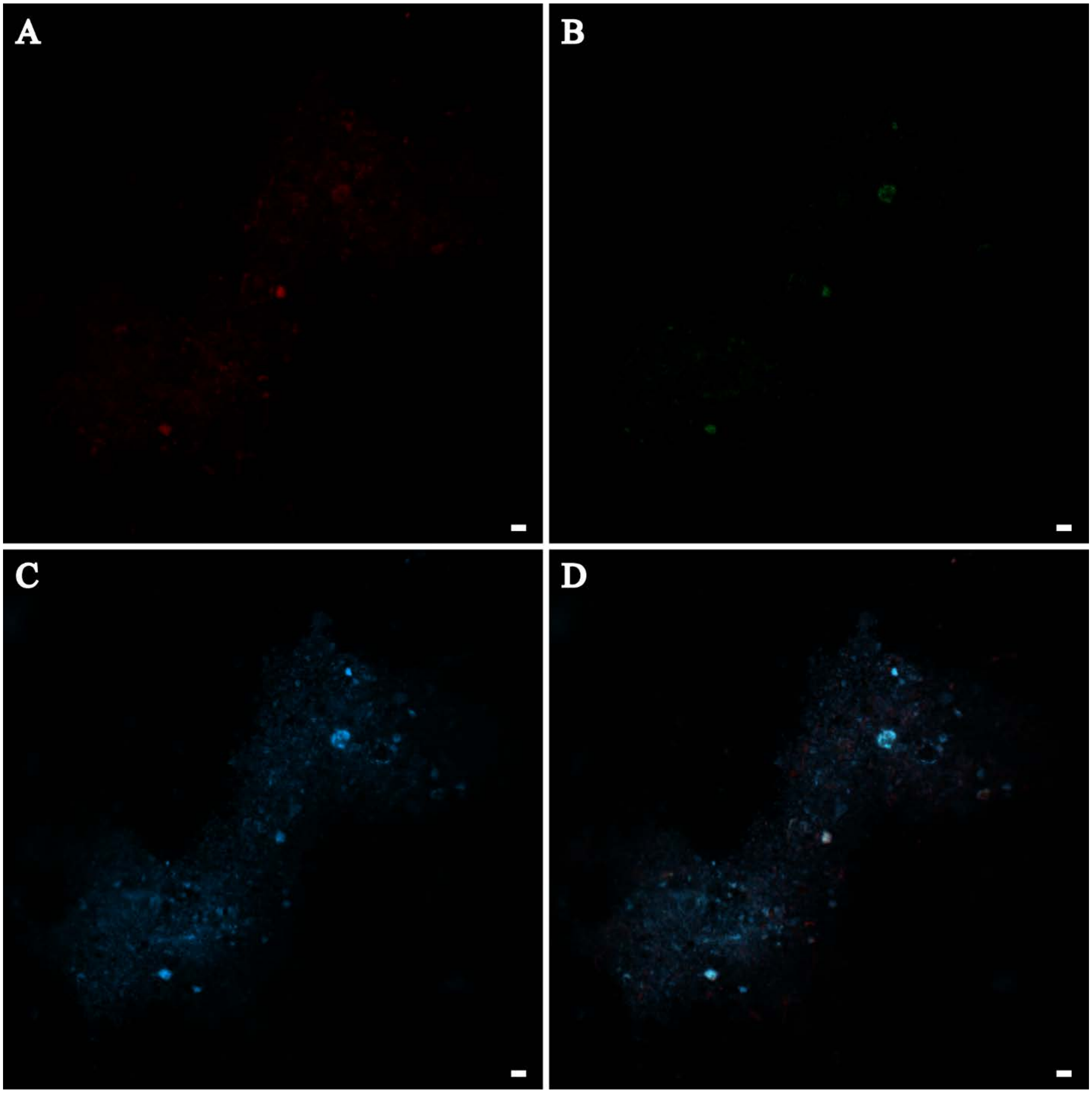

Supplementary Figure S2. Confocal laser scanning micrographs of the biofilm from GC1048 after applying the antisense probe NON338 labelled with 6-FAM as negative control visualized by FISH. Scale bars = 2 µm. **(A)** Signals of autofluorescence of particles or/and cells within the biofilm matrix - excitation of Cy3 [red]. **(B)** Signals of autofluorescence and unspecific probe binding of NON338 to particles or/and cells within the biofilm matrix - excitation of 6-FAM [green]. **(C)** Nucleic acids stained with DAPI [blue]. **(D)** Overlay of image A, B and C.
